# Supplementary material for: Degron-tagged reporters probe membrane topology and enable the specific labelling of membrane-wrapped structures
Source: Nat Commun. 2019 Aug 2;10:3490. doi: 10.1038/s41467-019-11442-z (PMC6677802; doi:10.1038/s41467-019-11442-z)
Supplement: Supplementary file 1 — Supplementary Information [file 41467_2019_11442_MOESM1_ESM.pdf]

Supplementary Information

Degron-tagged reporters probe membrane topology and enable the specific labelling of membrane-wrapped structures

KB Beer, G Fazeli et al.

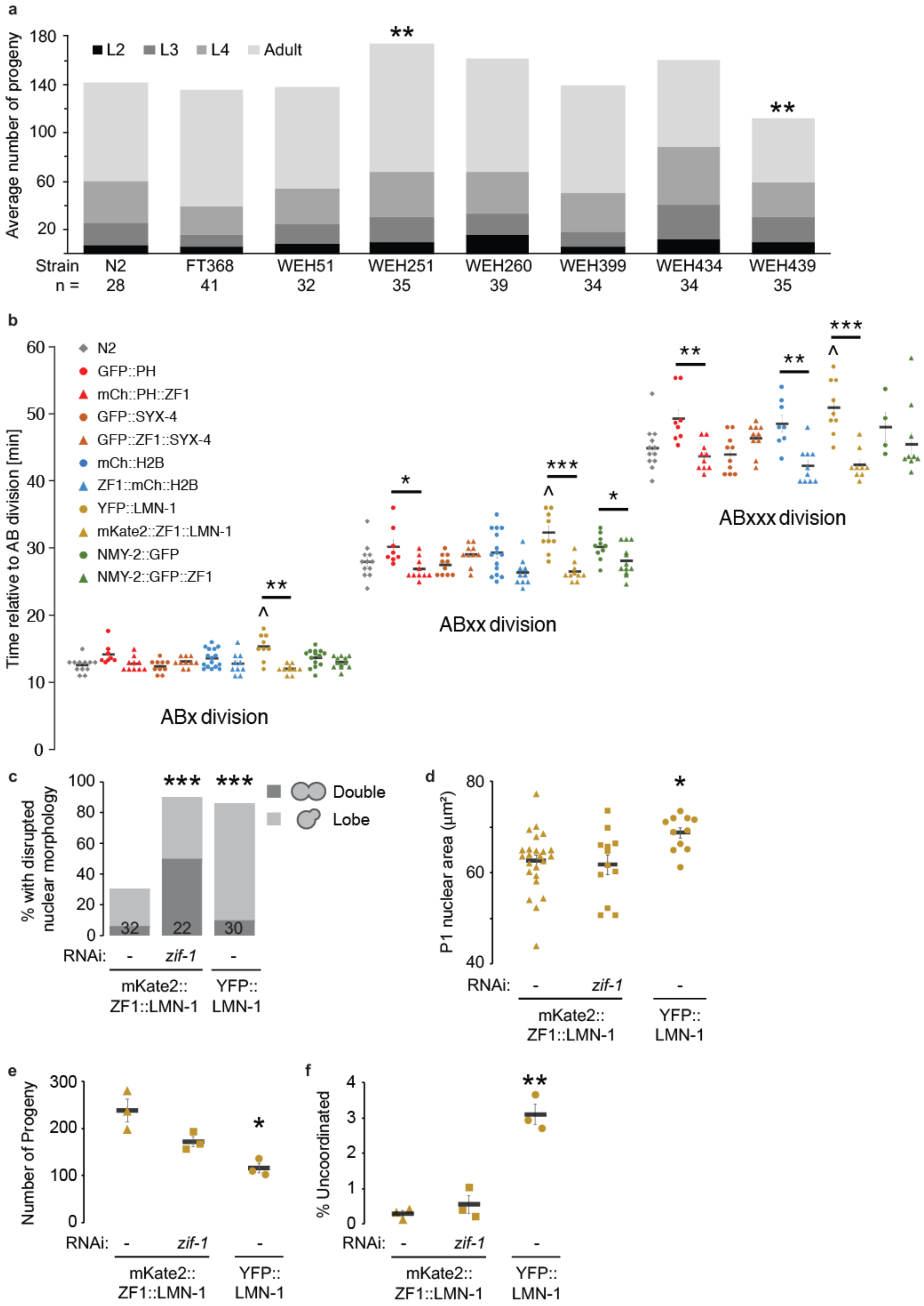
a
Average number of progeny
L2
L3
L4
Adult
Strain
n =
N2
28
FT368
41
WEH51
32
WEH251
35
WEH260
39
WEH399
34
WEH434
34
WEH439
35
b
Time relative to AB division [min]
N2
GFP::PH
mCh::PH::ZF1
GFP::SYX-4
GFP::ZF1::SYX-4
mCh::H2B
ZF1::mCh::H2B
YFP::LMN-1
mKate2::ZF1::LMN-1
NMY-2::GFP
NMY-2::GFP::ZF1
ABx division
ABxx division
ABxxx division
c
% with disrupted nuclear morphology
Double
Lobe
RNAi:
zif-1
mKate2::ZF1::LMN-1
YFP::LMN-1
d
P1 nuclear area (μm²)
e
Number of Progeny
f
% Uncoordinated

Supplementary Figure 1

Degron-tagged reporters do not generally disrupt development.

a) Degron-reporter strains showed no delay in long-term development (p>0.1, Chi-squared test). WEH251 (mKate2::ZF1::LMN-1) had significantly increased progeny in comparison to wild type worms (N2), while WEH439 expressing two degron-tagged reporters (ZF1::mCh::CHC-1; GFP::ZF1::PH) showed a significant reduction in total progeny (**p<0.01, Student's t-test with Bonferroni correction). The number of worms whose progeny was counted for each strain is indicated. Source data from a-f are provided as a Source Data file.

b) The timing of subsequent divisions of the anterior AB cell were compared between wild type (grey diamonds, n=12) and reporter strains with (triangles) or without the ZF1 degron (circles). Developmental timing was not significantly delayed after ZF1-tagging the PH domain of PLC1∂1 (red), the syntaxin SYX-4 (brown), a histone H2B (blue), Lamin LMN-1 (yellow), or non-muscle myosin NMY-2 (green) (p>0.1 using Student's t-test with Bonferroni correction). Developmental timing was delayed in a strain expressing YFP-tagged LMN-1 without a degron (^p<0.05). Division timing was slower in control fluorescent-tagged strains than their ZF1-tagged counterparts (PH: n=10 WEH260 vs. n=8 WEH02 embryos, SYX-4: n=10 FT368 vs. n=10 FT205, H2B: n=10 WEH296 vs. n=5 WEH142 or n=3-11 WEH248, LMN-1: n=9-10 WEH251 vs. n=9 XA3502, and NMY-2: n=10-12 WEH51 vs. n=4-14 BV113). Bars represent mean ± s.e.m. *p<0.05, **p<0.01, ***p<0.001.

c) Nuclear morphology was disrupted after LMN-1 overexpression. The penetrance of double nuclei or lobed nuclei increased after disrupting degron-mediated degradation of mKate2::ZF1::LMN-1 by *zif-1* RNAi (***p<0.001 using a two-tailed Fisher's exact test). The number of embryos examined are indicated.

d) The P1 nucleus was significantly enlarged in the YFP::LMN-1 strain XA3502 (n=11) compared to the degron-tagged strain with (n=12) or without *zif-1* RNAi (n=24). Bars in d-f represent mean ± s.e.m. (*p<0.05, **p<0.01 using Student's t-test with Bonferroni correction).

e) The total number of progeny was significantly decreased in the YFP::LMN-1 strain XA3502 compared to the degron-tagged strain with or without *zif-1* RNAi. n=3 replicates of 3 worms per plate.

f) The percentage of uncoordinated (Unc) worms was significantly increased in the YFP::LMN-1 strain XA3502 compared to the degron-tagged strain with or without *zif-1* RNAi. n=3 replicates of 3 worms per plate.

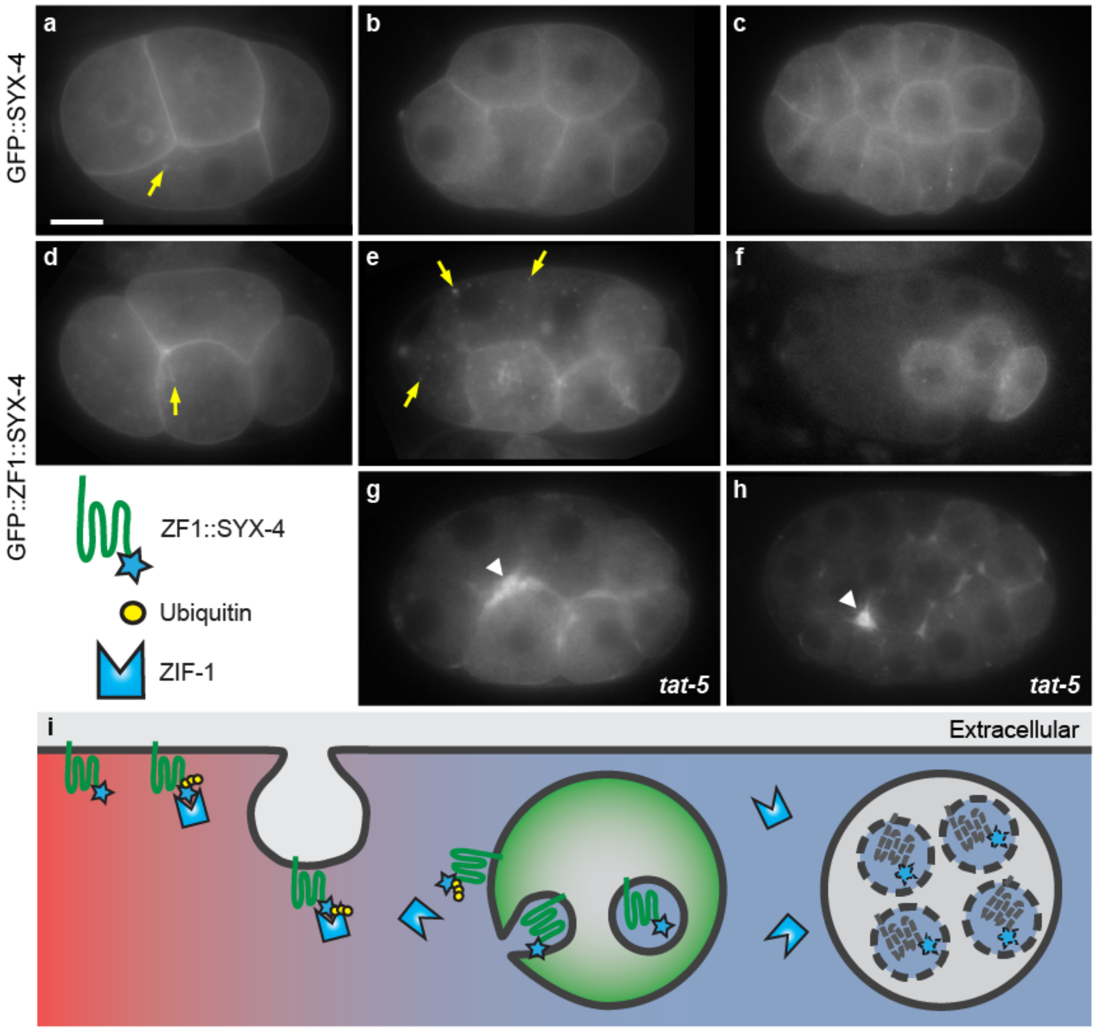

Supplementary Figure 2

Degron tags drive endocytosis and lysosomal degradation of a transmembrane reporter.

a-c) A transmembrane syntaxin localizes to the plasma membrane and endosomes (arrow) in 4-, 8-, and 26-cell GFP::SYX-4-expressing embryos (n=34). Scale bar: 10 µm. d) Prior to ZIF-1 expression, the degron-tagged GFP::ZF1::SYX-4 reporter localizes to the plasma membrane and endosomes (arrow). e) After ZIF-1 expression starts in anterior blastomere (AB) daughter cells, GFP::ZF1::SYX-4 is endocytosed (arrows) and is no longer found in the plasma membrane. f) After endocytosis, GFP::ZF1::SYX-4 vesicles disappear from the anterior cells by the 26-cell stage, consistent with acidification and degradation inside lysosomes (n=43). Anterior is left, dorsal is up. g-h) Embryos treated with *tat-5* RNAi show increased GFP::ZF1::SYX-4 membrane labelling due to accumulated microvesicles (arrowhead) at the 8- and 26-cell stage (n=29). i) A transmembrane protein tagged with the ZF1 degron is recognized by the ubiquitin ligase adaptor ZIF-1 at the plasma membrane and ubiquitinated. Polyubiquitination leads to endocytosis and microautophagy, which results in degradation of reporters on intraluminal vesicles within lysosomes (dotted lines) of degron-tagged transmembrane reporters.

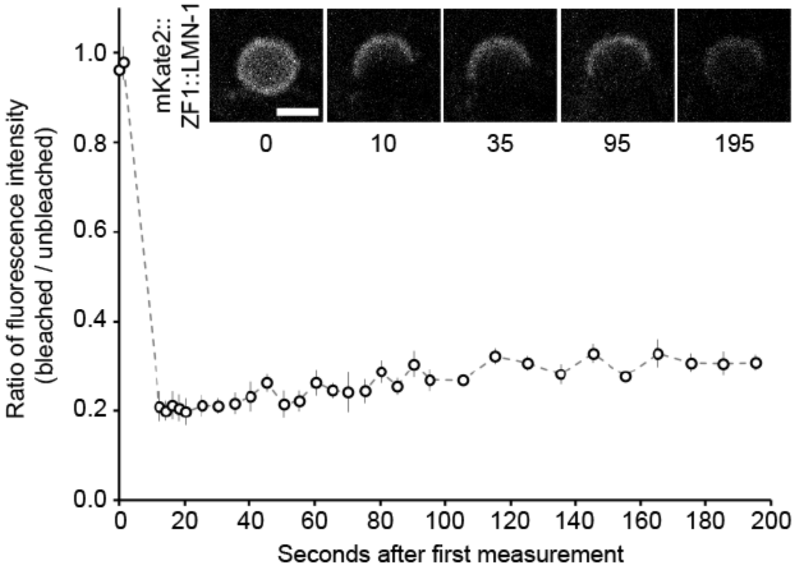

Supplementary Figure 3

LMN-1 remains immobile during degron-mediated degradation.

Half of the ABp nuclear lamina expressing mKate2::ZF1::LMN-1was photobleached during the 4-cell stage and imaged. The fluorescence of mKate2::ZF1::LMN-1 does not significantly recover after photobleaching ($p>0.3$ using Student's t-test with Bonferroni correction), indicating that its mobility is not increased after ubiquitination (n=3-5). Data are represented as mean ± s.e.m. Scale bar: 5 µm. Source data are provided as a Source Data file.

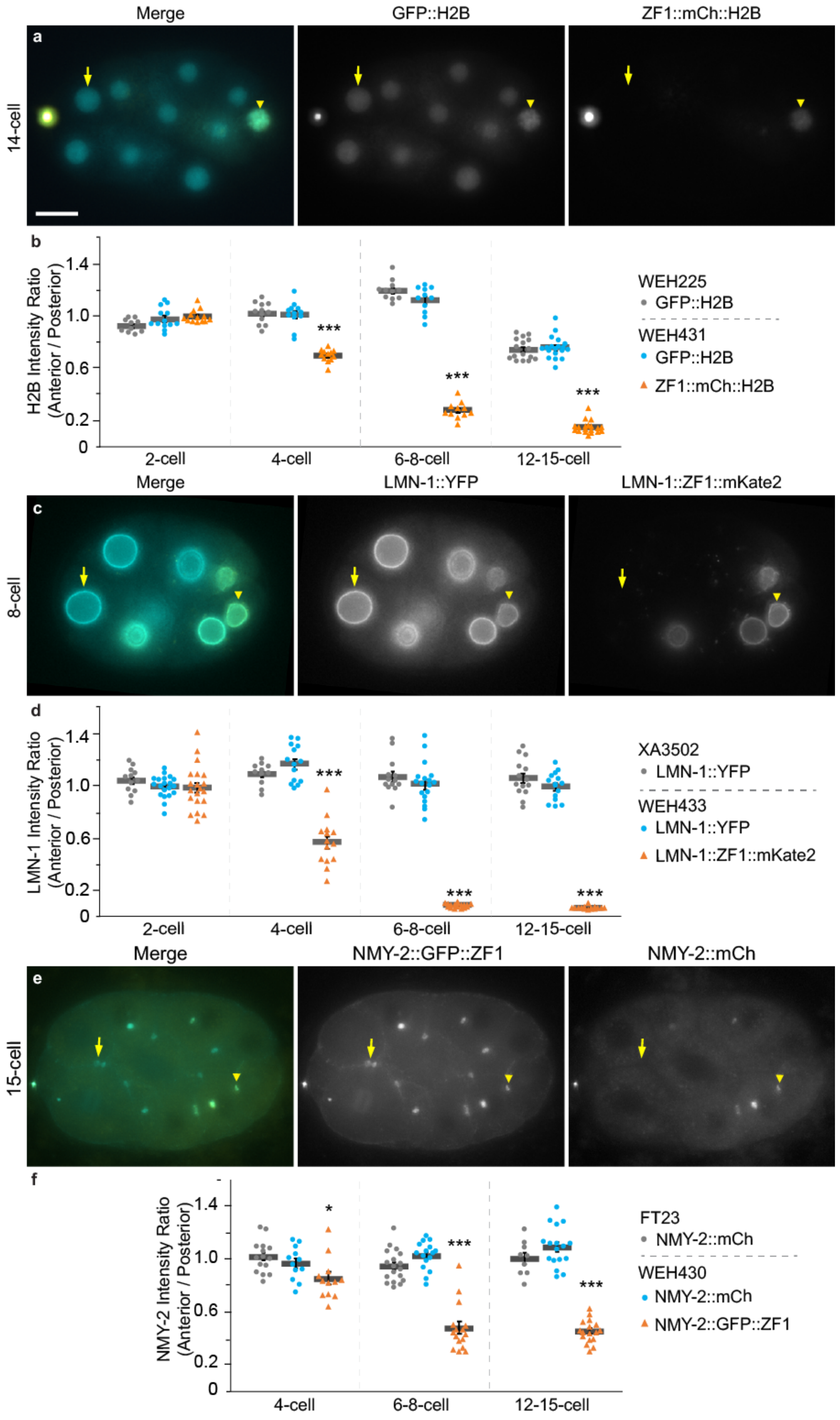

Merge
GFP::H2B
ZF1::mCh::H2B
a
14-cell
b
H2B Intensity Ratio (Anterior / Posterior)
1.4
1.0
0.6
0.2
0
***
2-cell
4-cell
6-8-cell
12-15-cell
WEH225
GFP::H2B
WEH431
GFP::H2B
ZF1::mCh::H2B
Merge
LMN-1::YFP
LMN-1::ZF1::mKate2
c
8-cell
d
LMN-1 Intensity Ratio (Anterior / Posterior)
XA3502
LMN-1::YFP
WEH433
LMN-1::YFP
LMN-1::ZF1::mKate2
Merge
NMY-2::GFP::ZF1
NMY-2::mCh
e
15-cell
f
NMY-2 Intensity Ratio (Anterior / Posterior)
*
FT23
NMY-2::mCh
WEH430
NMY-2::mCh
NMY-2::GFP::ZF1

Supplementary Figure 4

Degron-tagged reporters do not cause degradation of untagged binding partners.

a) The WEH431 strain expresses GFP-tagged H2B and ZF1 degron::mCherry-tagged H2B. Scale bar: 10 μm. b) The ratio of the fluorescence between anterior (somatic, arrow) and posterior (germ line, arrowhead) nuclei of the WEH431 strain (n=11-19) was compared with the ratio of the fluorescence of the WEH225 strain (n=10-16) expressing only GFP-tagged H2B at the same cell stage. ZF1::mCh::H2B drops significantly in anterior somatic cells starting during the 4-cell stage, but there is no significant change to GFP::H2B fluorescence in the presence of the degron-tagged H2B reporter ($p>0.1$ using Student's t-test with Bonferroni correction). Bars represent mean ± s.e.m. (*$p<0.05$, ***$p<0.001$). Source data are provided as a Source Data file.

c) The WEH433 strain expresses YFP-tagged LMN-1 and mKate2::ZF1 degron-tagged LMN-1. d) The ratio of the fluorescence between anterior (somatic, arrow) and posterior (germ line, arrowhead) nuclear lamina of the WEH433 strain (n=14-20) was compared with the ratio of the fluorescence of the XA3502 strain (n=11-14) expressing only YFP-tagged LMN-1 at the same cell stage from two independent experiments. mKate2::ZF1::LMN-1 drops significantly in anterior somatic cells from the 4-cell stage, but there is no significant change to YFP::LMN-1 in the presence of the degron-tagged LMN-1 reporter ($p>0.3$ using Student's t-test with Bonferroni correction). Bars represent mean ± s.e.m. (*$p<0.05$, ***$p<0.001$). Source data are provided as a Source Data file.

e) The WEH430 strain expresses mCherry-tagged NMY-2 and GFP::ZF1 degron-tagged NMY-2. f) The ratio of the fluorescence between anterior (somatic, arrow) and posterior (germ line, arrowhead) midbodies of the WEH430 strain (n=12-17) was compared with the ratio of the fluorescence of the FT23 strain (n=9-18) expressing only mCherry-tagged NMY-2 at the same cell stage. NMY-2::GFP::ZF1 drops significantly in anterior somatic cells starting during the 4-cell stage, but there is no significant change in NMY-2::mCherry in the presence of the degron-tagged NMY-2 reporter ($p>0.05$ using Student's t-test with Bonferroni correction). Bars represent mean ± s.e.m. (*$p<0.05$, ***$p<0.001$). Source data are provided as a Source Data file.

Supplementary Table 1: Strains used in this study.

Strain	Genotype	Source
AZ212	*unc-119(ed3) ruIs32[pAZ132: pie-1p::GFP::H2B; unc-119(+)] III*	Praitis et al., 2001[1]
BV113	*zuIs45[nmy-2::NMY-2::GFP, unc-119(+)] IV;*<br>*zbIs2[pie-1::Lifeact::mCherry, unc-119(+)]*	Singh & Pohl 2014[2]
DP38	*unc-119(ed3) III; daf-?*	CGC
FT23	*unc-119(ed3) III;*<br>*xnIs8 [pJN343: nmy-2::NMY-2::mCherry + unc-119(+)]*	Nelson et al., 2011[3]
FT166	*xnIs65 [nmy-2::gfp::zf1 + unc-119(+)]; unc-119(ed3) III*	Fazeli et al., 2016[4]
FT205	*unc-119(ed3) III;*<br>*xnIs88[MP322: syx-4::GFP::SYX-4::syx-4 3'UTR, unc-119(+)];*	This study
FT368	*unc-119(ed3) III;*<br>*xnIs144[syx-4::GFP::ZF1::SYX-4::syx-4 3'UTR, unc-119(+)]*	Wehman et al., 2011[5]
FT1091	*unc-119(ed3) III;*<br>*xnIs390[pie-1::GFP::ZF1::PH(PLC1delta1), unc-119(+)]*	Beer et al., 2018[6]
FT1217	*chc-1(ok2369) III / hT2[bli-4(e937) let-?(q782) qIs48] (I; III);*<br>*xnIs388[pJN578: chc-1p::ZF1::mCherry::chc-1, unc-119(+)]*	Gift of Jeremy Nance
HT1593	*unc-119(ed3) III*	CGC
N2	Wild type	Brenner, 1974[7]
OD301	*unc-119(ed3) III;*<br>*ltIs76[pAA178: pie-1::mCherry::SP12; unc-119 (+)]*	Gift of Karen Oegema
WEH02	*unc-119(ed3) III; ltIs38[pie-1::GFP::PH(PLC1∂1) + unc-119(+)];*<br>*xnIs8[pJN343: nmy-2::NMY-2::mCherry, unc-119(+)]*	Fazeli et al., 2016[4]
WEH51	*unc-119(ed3) III; xnIs65[nmy-2::gfp::zf1, unc-119(+)] IV;*<br>*ltIs44[pie-1::mCherry::PH(PLC1∂1), unc-119(+)] V*	Fazeli et al., 2016[4]
WEH132	*unc-59(e261) I; unc-119(ed3) III;*<br>*xnIs65[nmy-2::gfp::zf1, unc-119(+)] IV;*<br>*ltIs44[pie-1::mCherry::PH(PLC1∂1), unc-119(+)] V*	Fazeli et al., 2016[4]
WEH142	*unc-119(ed3) III; zbIs1[pie-1::Lifeact::GFP, unc-119(+)];*<br>*Is[mCherry::HistoneH2B] IV*	Fazeli et al., 2018[8]
WEH225	*Is[pVIG57: Pmex-5::mCherry::LGG-2::tbb-2 3'UTR,*<br>*C.b. unc-119(+)] II;*<br>*unc-119(ed3) ruIs32[pAZ132: pie-1::GFP::H2B, unc-119(+)] III*	Fazeli et al., 2018[8]
WEH248	*unc-119(ed3) III; pwIs21[pie-1::gfp::rab-7, unc-119(+)];*<br>*Is[mCherry::HistoneH2B] IV*	Fazeli et al., 2018[8]
WEH251	*unc-119(ed3) III;*<br>*wurIs85[pGF04: pie-1::mKate2::ZF1::LMN-1, pJN254: unc-119(+)]*	This study
WEH260	*unc-119(ed3) III;*<br>*wurIs90[pGF7: pie-1::mCherry::PH(PLC1∂1)::ZF1, unc-119(+)]*	Beer et al., 2018[6]
WEH296	*unc-119(ed3) III;*<br>*+ / wurIs103[pGF13: pie-1::ZF1::mCherry::his-15, unc-119(+)]*	Fazeli et al., 2018[8]
WEH399	*unc-119(ed3) III;*<br>*wurIs144[pGF13: pie-1::ZF1::mCherry::his-15, unc-119(+)]*	This study
WEH430	*unc-119(ed3) xnIs8[pJN343: nmy-2::NMY-2-mCherry; unc-119(+)*<br>*line 26B] III; xnIs65 (nmy-2-gfp-zf1, unc-119) IV*	Cross FT23 x FT166

WEH431	*unc-119(ed3) ruIs32[pAZ132: pie-1::GFP::H2B, unc-119(+)] III; wurIs144[pGF13: pie-1::ZF1::mCherry::his-15; unc-119(+)]*	Cross AZ212 x WEH399
WEH433	*unc-119(ed3) III;*<br>*qaIs3502[unc-119(+) pie-1::YFP::lmn-1, pie-1::CFP::H2B];*<br>*wurIs85[pGF04: pie-1::mKate2::ZF1::LMN-1, pJN254: unc-119(+)]*	Cross WEH251 x XA3502
WEH434	*unc-119(ed3) III; wurIs155[pAZ132-coPH-oma-1(219-378): pie-1::mCh::PH::CTPD, unc-119(+)]*	This study
WEH439	*unc-119(ed3) III;*<br>*xnIs388[pJN578: chc-1p::ZF1::mCh::chc-1, unc-119(+)];*<br>*xnIs390[pie-1::GFP::ZF1::PH(PLC1delta1), unc-119(+)]*	Cross FT1091 x FT1217
WEH447	*unc-119(ed3) III; wurIs161 [ss-pCFJ1954-ZF1-KDEL: eft-3p:: sel-1ss::tagRFP-T::ZF1::KDEL::tbb-2 3'UTR, C.b. unc-119(+)]*	This study
XA3502	*unc-119(ed3) III;*<br>*qaIs3502[unc-119(+), pie-1::YFP::lmn-1, pie-1::CFP::H2B]*	Galy et al., 2003[9]

Supplementary Table 2: Primers used in this study.

Name	Sequence
oma-1(378) Stop attL2 F	ctcatgaaatctgtcgcaactgattaaaacccagctttcttgtacaaagt
oma-1(219) coPH R	ggcagaaagtgggggagcggcaatcttttgacgttggtccatggat
coPH oma-1(219) F	ggatccatggaccaacgtcaaaagattgccgctcccccactttctgccat
attL2 Stop oma-1(378) R	gtacaagaaagctgggttttaatcagttgcgacagatttcatgagaa
sel-1 ss flex F	cacctccgccacctgccaaaagaagtccgccaccggatcatcctccacatcatcctcca
sel-1 ss eft-3p R	gcgagtagcaacagtgtcaggtaggttttaatcatggctgctacggagtgagcaa
coZF1 KDEL Stop attB2 F	cacctacgcccacggacaagacgagctccgtgtcccaagatccggaactaaggacgagctctaaaaccca gctttcttgtacaaagtgg
coZF1 flex R	cagttgtcgttgtatgggcagtatccctcacgacggaaggcgtcgcagagacgggtcttgtactcggtggctg gggaagtaccggatga

Supplementary Methods

Progeny counts

Single L4 worms from wild type N2 and degron reporter strains were singled onto three 12-well plates. The wells were photographed 3.75 days later on a Leica M80 with a Leica MC120 HD camera. Juvenile L2-L4 and adult progeny were counted using the Cell Counter in Fiji (NIH). For comparison of LMN-1 reporter strains, three L4 XA3502 worms or WEH251 with or without *zif-1* RNAi treatment were put on one 60 mm plate and progeny were counted 3.75 days later. The number of worms with motility deficits (Unc) was reported as a percentage of the total worms counted on each plate. Wells where the mother died (n=10) or the worms clumped (n=12) were excluded from progeny counts.

Cell cycle timing

To compare the speed of development between control and ZF1-tagged strains, the time from the 3- to the 6-, 12-, and 24-cell stages (one to three subsequent cell cycles in the AB lineage) were calculated from time-lapse series. The time of the AB cell division (3-cell stage) was compared to the timing of the AB daughter (ABx, 6-cell), AB granddaughter (ABxx, 12-cell) and AB great-granddaughter (ABxxx, 24-cell) cell divisions. The cell cycle time was measured from cytokinetic furrow ingression in the AB cell to the ingression of any furrow in the two ABx, four ABxx or eight ABxxx cells. One embryo from FT205 strain was excluded because of cytokinesis defect.

Quantification of nuclei size

The widest cross-section was used to calculate the nuclear area prior to AB cell division. The edge of the P1 nuclear lamina was traced using Fiji (NIH) from fluorescent images of XA3502 and WEH251, as well as WEH251 fed with *zif-1* RNAi.

Fluorescence recovery after photobleaching

Fluorescence recovery after photobleaching (FRAP) was conducted using a Leica SP8 confocal with a HCX PL APO CS 40.0x1.25-NA oil objective with PMT detectors. Half of the ABp nucleus was bleached using the 560 nm laser line. Images were acquired before and after bleaching with intervals of 2 seconds (5 times), 5 seconds (15 times), and 10 seconds (10 times). Fluorescence intensity was measured in three areas of 0.5 μm^2 on the nuclear envelope within the bleached region and three areas within the nonbleached region using ImageJ (NIH). Data are reported as the ratio of the average intensity in bleached regions to the average intensity in nonbleached regions. Embryos were excluded when focus was lost very early during imaging (n=3). In 2 movies, focus was lost during the last two minutes. Those time points were not included in measurements.

Fluorescence intensity measurements

The mean fluorescence of H2B reporters (supplementary figure 4b) was measured in a circle with an area of 2.6 μm^2 in the center of interphase nuclei from 2- to 15-cell stage embryos using Fiji (NIH). In older embryos

where ZF1::mCh::H2B was no longer visible in AB nuclei, GFP::H2B was used to find the nucleus. Data is reported as a ratio of the mean fluorescence of the anterior AB lineage nuclei to the posterior P lineage nuclei. Images were excluded when AB or P nuclei had condensed mitotic chromosomes (n=29).

Mean fluorescence intensity of LMN-1 reporters for supplementary figure 4d was measured using a 3 μm line two pixels in width using Fiji (NIH). The line was drawn at the brightest region of the nuclear membrane from images of 2- to 15-cell stage embryos. The intensity was measured in anterior AB cells and posterior P cells, except when P cells were dividing, in which case P sister cells, including E at the 7-cell stage (n=1) or C at the 14-cell stage (n=1) were measured. In older embryos where ZF1-tagged LMN-1was no longer visible in AB nuclei, YFP::LMN-1 was used to trace the nucleus. Embryos were excluded during NEBD of ABx (n=5) or when the nuclei morphology was extremely malformed (n=4) or when the cell identity could not be determined (n=1).

Mean fluorescence intensity of NMY-2 reporters (supplementary figure 4f) was measured in a circle with an area of 1 μm^2 in midbodies after abscission in 2- to 15-cell embryos using Fiji (NIH). Data is presented as a ratio of the fluorescence intensity of anterior midbodies to posterior midbodies. In older embryos where ZF1-tagged NMY-2 was no longer visible in AB nuclei, NMY-2::mCh was used to locate the midbody. Images were excluded when midbodies were out of focus (n=3) or endocytosed (n=20) or when the cell identity could not be determined (n=1).

Supplementary References

1. Praitis, V., Casey, E., Collar, D. & Austin, J. Creation of low-copy integrated transgenic lines in Caenorhabditis elegans. *Genetics* **157,** 1217–1226 (2001).
2. Singh, D. & Pohl, C. Coupling of Rotational Cortical Flow, Asymmetric Midbody Positioning, and Spindle Rotation Mediates Dorsoventral Axis Formation in C. elegans. *Dev. Cell* **28,** 253–267 (2014).
3. Nelson, M. D. *et al.* A bow-tie genetic architecture for morphogenesis suggested by a genome-wide RNAi screen in Caenorhabditis elegans. *PLoS Genet.* **7,** e1002010 (2011).
4. Fazeli, G., Trinkwalder, M., Irmisch, L. & Wehman, A. M. C. elegans midbodies are released, phagocytosed and undergo LC3-dependent degradation independent of macroautophagy. *J. Cell Sci.* **129,** 3721–3731 (2016).
5. Wehman, A. M., Poggioli, C., Schweinsberg, P., Grant, B. D. & Nance, J. The P4-ATPase TAT-5 inhibits the budding of extracellular vesicles in C. elegans embryos. *Curr. Biol.* **21,** 1951–1959 (2011).
6. Beer, K. B. *et al.* Extracellular vesicle budding is inhibited by redundant regulators of TAT-5 flippase localization and phospholipid asymmetry. *Proc. Natl. Acad. Sci.* **115,** E1127–E1136 (2018).
7. Brenner, S. The genetics of Caenorhabditis elegans. *Genetics* **77,** 71–94 (1974).
8. Fazeli, G., Stetter, M., Lisack, J. N. & Wehman, A. M. C. elegans Blastomeres Clear the Corpse of the Second Polar Body by LC3-Associated Phagocytosis. *Cell Rep.* **23,** 2070–2082 (2018).
9. Galy, V., Mattaj, I. W. & Askjaer, P. Caenorhabditis elegans Nucleoporins Nup93 and Nup205 Determine the Limit of Nuclear Pore Complex Size Exclusion In Vivo. *Mol. Biol. Cell* **14,** 5104–5115 (2003).
