# Supplementary material for: Degron-tagged reporters probe membrane topology and enable the specific labelling of membrane-wrapped structures
Source: Nat Commun. 2019 Aug 2;10:3490. doi: 10.1038/s41467-019-11442-z (PMC6677802; doi:10.1038/s41467-019-11442-z)
Supplement: Supplementary file 3 — Description of Additional Supplementary Files [file 41467_2019_11442_MOESM3_ESM.pdf]

Description of Additional Supplementary Files

File name: Supplementary Movie 1

Description: ZF1 degron reporters facilitate detection of released extracellular vesicles and polar bodies. Time-lapse movies of WEH260 embryos expressing mCh::PH::ZF1 on membranes (cyan) with or without *tat-5* RNAi treatment to induce extracellular vesicle accumulation. In an untreated control embryo, mCh::PH::ZF1 localizes to the plasma membrane, but gradually disappears in somatic cells, starting with the anterior cells. In a *tat-5* RNAi-treated embryo, mCh::PH::ZF1 persists on released extracellular vesicles that accumulate between cells. mCh::PH::ZF1 persists in posterior germ cells, as well as in polar bodies in both control and *tat-5* RNAi-treated embryos. Anterior is to the left, dorsal is up. Time-lapse data were collected every minute for 90 minutes (6 fps). 9 Zs are projected with an interval of 1.2 μm.

File name: Supplementary Movie 2

Description: CTPD degron reporters facilitate detection of released extracellular vesicles and polar bodies. Time-lapse movies of WEH434 embryos expressing mCh::PH::CTPD on membranes (cyan) with or without *tat-5* RNAi treatment to induce extracellular vesicle accumulation. In an untreated control embryo, mCh::PH::CTPD localizes to the plasma membrane, but gradually disappears during the first cell division. In a *tat-5* RNAi-treated embryo, mCh::PH::CTPD persists on released extracellular vesicles that accumulate between cells. mCh::PH::CTPD persists in polar bodies in both control and *tat-5* RNAi-treated embryos. Anterior is to the left, dorsal is up. Time-lapse data were collected every 40 seconds for 27 minutes (6 fps). 12 Zs are projected for the control embryo with an interval of 1 μm, while 10 Zs are projected for the *tat-5(RNAi)* embryo with an interval of 1.2 μm.

File name: Supplementary Movie 3

Description: Degron reporters enable tracking of a phagocytosed cell corpse. Time-lapse movies of WEH142 embryos expressing mCh::H2B and WEH339 embryos expressing ZF1::mCh::H2B in nuclei, including both polar bodies (arrowheads). It is harder to follow the corpse of the second polar body labelled with mCh::H2B, due to the fluorescence of nearby nuclei. ZF1::mCh::H2B is degraded in sequential sets of somatic nuclei, facilitating tracking of the polar bodies as they remain the only fluorescent structures in the anterior half of the embryo. Anterior is to the left, dorsal is up, and the embryos are within the dotted oval. Time-lapse data were collected every 20 seconds for WEH142 and every 30 seconds for WEH339 for 40 minutes in total (playing at 15 fps and 10 fps, respectively). 11 Zs with 1.2 μm intervals are projected.

File name: Supplementary Movie 4

Description: Degron reporters reveal nuclear envelope dynamics during cell division. Time-lapse movies of WEH251 embryos expressing mKate2::ZF1::LMN-1 to mark the nuclear lamina. In control embryos, the reporter gradually loses fluorescence intensity in anterior somatic cells during interphase, followed by a sharp drop of fluorescence in dividing cells when nuclear envelope breakdown occurs. When the ubiquitin ligase adaptor ZIF-1 is knocked down, the mKate2::ZF1::LMN-1 fluorescence is stable in all cells. Anterior is to the left, dorsal is up. Time-lapse data were collected every 20 seconds for 21-22 minutes (6 fps). 8 Zs with 1 μm intervals are projected.

File name: Supplementary Movie 5

Description: LMNA is protected from NES-TIR1 during interphase. Representative time-lapse series of a HeLa cell transiently expressing NES-TIR1 and Venus-mAID-LMNA. 0.5 mM NAA was added at t = 0 to induce auxin-dependent degradation. During interphase, Venus-mAID-LMNA in the nuclear lamina is protected from ubiquitination by cytosolic NES-TIR1 and fluorescence persists in the presence of NAA. Data were collected every 5 minutes. Scale bar: 30 μm.

File name: Supplementary Movie 6

Description: LMNA is accessible to NES-TIR1 during mitosis. Representative time-lapse series of a HeLa cell transiently expressing NES-TIR1 and Venus-mAID-LMNA entering mitosis (same cell as Video 5). After nuclear envelope breakdown (NEBD, t = 0), Venus-mAID-LMNA is accessible by cytosolic NES-TIR1 and fluorescence rapidly disappears. Data were collected every 5 minutes. Scale bar: 30 μm.

File name: Supplementary Movie 7

Description: LMNA is accessible to NLS-TIR1 during interphase. Interphase HeLa cell stably expressing NLS-TIR1 and NES-TIR1 from a single mRNA in addition to Venus-mAID-LMNA. After treatment with 0.5 mM NAA (t = 0), Venus-mAID-LMNA fluorescence disappears during interphase. Data were collected every 5 minutes. Scale bar: 30 μm.
