# Supplementary material for: Degron-tagged reporters probe membrane topology and enable the specific labelling of membrane-wrapped structures
Source: Nat Commun. 2019 Aug 2;10:3490. doi: 10.1038/s41467-019-11442-z (PMC6677802; doi:10.1038/s41467-019-11442-z)
Supplement: Supplementary file 11 — Reporting Summary [file 41467_2019_11442_MOESM11_ESM.pdf]

nature research

Corresponding author(s): Ann M. Wehman

Reporting Summary

Nature Research wishes to improve the reproducibility of the work that we publish. This form provides structure for consistency and transparency in reporting. For further information on Nature Research policies, see Authors & Referees and the Editorial Policy Checklist.

Statistical parameters

When statistical analyses are reported, confirm that the following items are present in the relevant location (e.g. figure legend, table legend, main text, or Methods section).

n/a	Confirmed	
☐	☒	The exact sample size (n) for each experimental group/condition, given as a discrete number and unit of measurement
☐	☒	An indication of whether measurements were taken from distinct samples or whether the same sample was measured repeatedly
☐	☒	The statistical test(s) used AND whether they are one- or two-sided<br>*Only common tests should be described solely by name; describe more complex techniques in the Methods section.*
☒	☐	A description of all covariates tested
☐	☒	A description of any assumptions or corrections, such as tests of normality and adjustment for multiple comparisons
☐	☒	A full description of the statistics including central tendency (e.g. means) or other basic estimates (e.g. regression coefficient) AND variation (e.g. standard deviation) or associated estimates of uncertainty (e.g. confidence intervals)
☒	☐	For null hypothesis testing, the test statistic (e.g. F, t, r) with confidence intervals, effect sizes, degrees of freedom and P value noted<br>*Give P values as exact values whenever suitable.*
☒	☐	For Bayesian analysis, information on the choice of priors and Markov chain Monte Carlo settings
☒	☐	For hierarchical and complex designs, identification of the appropriate level for tests and full reporting of outcomes
☒	☐	Estimates of effect sizes (e.g. Cohen's d, Pearson's r), indicating how they were calculated
☐	☒	Clearly defined error bars<br>*State explicitly what error bars represent (e.g. SD, SE, CI)*

Our web collection on statistics for biologists may be useful.

Software and code

Policy information about availability of computer code

Data collection	Imaging was performed with Zeiss Axiovision software and Leica LAS AF software
Data analysis	Data sets were analysed using ImageJ or FIJI (NIH), Imaris (Bitplane), Prism 6 (GraphPad), and OriginPro (OriginLab)

For manuscripts utilizing custom algorithms or software that are central to the research but not yet described in published literature, software must be made available to editors/reviewers upon request. We strongly encourage code deposition in a community repository (e.g. GitHub). See the Nature Research guidelines for submitting code & software for further information.

Data

Policy information about availability of data

All manuscripts must include a data availability statement. This statement should provide the following information, where applicable:
- Accession codes, unique identifiers, or web links for publicly available datasets
- A list of figures that have associated raw data
- A description of any restrictions on data availability

The data sets generated during and/or analysed during the current study are available from the corresponding author on request.

Field-specific reporting

Please select the best fit for your research. If you are not sure, read the appropriate sections before making your selection.

☒ Life sciences ☐ Behavioural & social sciences ☐ Ecological, evolutionary & environmental sciences

For a reference copy of the document with all sections, see nature.com/authors/policies/ReportingSummary-flat.pdf

Life sciences study design

All studies must disclose on these points even when the disclosure is negative.

Sample size	No sample-size calculation was performed. Experiments were repeated on at least three independent sets of embryos. For individual images, n=20 embryos/set were examined to give a 95% confidence level. For time-lapse data sets, n=10 embryos was sought to give a 90% confidence level. Statistical analysis was performed on all measurements to test whether observed differences were significant.
Data exclusions	In Fig. 8, cells that died during imaging were excluded from analysis. As transient transfections with NES-TIR1 and Venus-mAID-LMNA did not result in transfection of all cells with both constructs, only cells that expressed Venus-mAID-LMNA and showed degradation after NEBD were considered as double positive and considered for analysis (Fig. 8a, b, d, f).<br>In Fig. 9g, embryos who arrested development during time-lapse imaging were excluded from measurements (n=3). Embryos were also excluded if the fluorescence from the P0 and AB midbody remnants merged during the time-lapse series (n=4) or if the polar body data did not fit an exponential decay function (n=1). Embryos treated with tsg-101 RNAi were excluded if the AB midbody remnant internalized by the 6-cell stage (n=6), as the RNAi was judged ineffective if it did not delay internalization.<br>In Fig. 10, embryos were excluded when the cell identity could not be determined (n=7).<br>In Fig. S1a, wells were excluded from the progeny counts when the mother died (n=10) or the worms clumped (n=12). In Fig. S1b, one embryo from the FT205 strain was excluded because of a cytokinesis defect.<br>In Fig. S3, embryos were excluded when focus was lost early during imaging (n=3). In 2 other movies, focus was lost during the last two minutes. Those time points were not included in measurements.<br>In Fig. S4b, embryos were excluded when AB or P nuclei had condensed mitotic chromosomes (n=29). In Fig. S4d, embryos were excluded during NEBD of ABx (n=5) or when the nuclei morphology was extremely malformed (n=4) or when the cell identity could not be determined (n=1). In Fig. S4f, embryos were excluded when midbodies were out of focus (n=3) or endocytosed (n=20) or when the cell identity could not be determined (n=1).
Replication	Experiments were repeated on at least three independent sets of embryos on multiple days, except for Fig. 4, 8g, and supplementary Fig. 4d. Most experiments were performed by at least two scientists.
Randomization	No randomization was performed as the animals were raised similarly under controlled laboratory conditions.
Blinding	Blinding was not attempted as the different genotypes or RNAi treatments give distinct results.

Reporting for specific materials, systems and methods

Materials & experimental systems

n/a	Involved in the study	
☐	☒	Unique biological materials
☒	☐	Antibodies
☐	☒	Eukaryotic cell lines
☒	☐	Palaeontology
☐	☒	Animals and other organisms
☒	☐	Human research participants

Methods

n/a	Involved in the study	
☒	☐	ChIP-seq
☒	☐	Flow cytometry
☒	☐	MRI-based neuroimaging

Unique biological materials

Policy information about availability of materials

Obtaining unique materials	All worm strains, cell lines, and plasmids used in this study are available from the authors or the sources indicated in the methods or supplemental table.

Eukaryotic cell lines

Policy information about cell lines

Cell line source(s)	HeLa Kyoto and HeLa FRT/TO cells were a gift from Jonathon Pines (ICR, London, UK).
Authentication	None of the cell lines were authenticated.
Mycoplasma contamination	All cell lines tested negative for mycoplasma.
Commonly misidentified lines (See ICLAC register)	No misidentified cell lines were used.

Animals and other organisms

Policy information about studies involving animals; ARRIVE guidelines recommended for reporting animal research

Laboratory animals	Caenorhabditis elegans hermaphrodite worms. See strain list and figure legends for full details.
Wild animals	The study did not involve wild animals.
Field-collected samples	The study did not involve samples collected from the field.
